# Lack of interchangeability between visual analogue and verbal rating pain scales: a cross sectional description of pain etiology groups

**DOI:** 10.1186/1471-2288-5-31

**Published:** 2005-10-04

**Authors:** Iréne Lund, Thomas Lundeberg, Louise Sandberg, Cecilia Norrbrink Budh, Jan Kowalski, Elisabeth Svensson

**Affiliations:** 1 Department of Physiology and Pharmacology, Karolinska Institutet, Stockholm, SE-171 77 Sweden.; 2 Rehabilitation Medicine Clinic, Danderyds Hospital AB, Stockholm, SE-182 88 Sweden.; 3 Spinalis SCI unit, Karolinska University Hospital, Stockholm, SE-169 89 Sweden.; 4 Department of Statistics (ESI), Örebro University, Örebro, SE 701-81 Sweden.

## Abstract

**Background:**

Rating scales like the visual analogue scale, VAS, and the verbal rating scale, VRS, are often used for pain assessments both in clinical work and in research, despite the lack of a gold standard. Interchangeability of recorded pain intensity captured in the two scales has been discussed earlier, but not in conjunction with taking the influence of pain etiology into consideration.

**Methods:**

In this cross-sectional study, patients with their pain classified according to its etiology (chronic/idiopathic, nociceptive and neuropathic pain) were consecutively recruited for self-assessment of their actual pain intensity using a continuous VAS, 0–100, and a discrete five-category VRS. The data were analyzed with a non-parametric statistical method, suitable for comparison of scales with different numbers of response alternatives.

**Results:**

An overlapping of the VAS records relative the VRS categories was seen in all pain groups. Cut-off positions for the VAS records related to the VRS categories were found lower in patients with nociceptive pain relative patients suffering from chronic/idiopathic and neuropathic pain. When comparing the VAS records transformed into an equidistant five-category scale with the VRS records, systematic disagreements between the scales was shown in all groups. Furthermore, in the test-retest a low percentage of the patients agreed to the same pain level on the VAS while the opposite hold for the VRS.

**Conclusion:**

The pain intensity assessments on VAS and VRS are in this study, not interchangeable due to overlap of pain records between the two scales, systematic disagreements when comparing the two scales and a low percentage intra-scale agreement. Furthermore, the lower VAS cut-off positions relative the VRS labels indicate different meaning of the rated pain intensity depending on pain etiology. It is also indicated that the scales have non-linear properties and that the two scales probably have different interpretation. Our findings are in favor of using the VRS in pain intensity assessments but if still the VAS is preferred, the VAS data should be analyzed as continuous using statistical methods suitable for ordinal data. Furthermore, our findings indicate a risk to over or under estimate the patient's perceived pain when interpreting condensed VAS data.

## Background

The assessment of perceived pain is necessary in the clinical setting for diagnosis and choice of treatment but also for the evaluation of treatment efficacy in a research context. The multidimensional pain sensation involves the subjective evaluations of the sensory aspect like intensity, the affective component such as unpleasantness and the cognitive aspect like thoughts related to the condition. The pain intensity, also mentioned as the severity of pain, is probably the most commonly assessed dimension of pain [1].

The level of personal pain experience is only possible to determine indirectly by self-reported ratings often by using uni-dimensional pain rating scales that may be used for various dimensions of pain. The most commonly used scales, both in ordinary clinical work and in research, are the continuous visual analogue scale, VAS, and discrete categorical scales like the verbal rating scale, VRS, and the numerical rating scale, NRS. Although widely used, there is so far no support for a rational choice of one of these scales [2] even though NRS has previously been recommended as an outcome measure for chronic/idiopathic pain clinical trials [3]. In the absence of gold standard there is a need to study to what extent the individual scores captured on one pain scale are interchangeable with the individual scoring on another pain scale, i.e. the quality of the intra-individual assessments.

On group level, the pain assessments on VAS and VRS have been variably reported as highly inter-correlated [4,5] but also as not being interchangeable [6,7] for example due to overlapping VAS records when related to the categories of the VRS. This overlap is obvious, albeit not highlighted, from the results of several studies related to various clinical conditions [8-10] though not in conjunction with taking the etiology or mechanism of pain classification into consideration. A similar overlap was also demonstrated when comparing VAS and NRS of pain in rest and during activity in different pain conditions [11].

To provide a rational treatment approach, classification of pain is also recommended according to its etiology [12,13] or, if possible, to its mechanism [14]. Since the pain experience is uncertainly related to the extent of injury or stimulation [15], the perceived pain may have linear or non-linear properties [16].

The purpose of this study was to evaluate the quality of the intra-individual assessments of self-reported pain intensity on a continuous VAS (0–100) and a discrete five-category VRS, in patients with pain. The patients were separately described in groups of pain etiology. The evaluation includes inter-scale concordance, implying to which extent the assessment on one scale can be replaced by the assessment on the other, without change of the result. The consistency between the scales were also evaluated when continuous VAS assessments were transformed into discrete scales defined by equidistant cut-off positions as well as by unbiased cut-off positions relative the VRS data. The intra-individual assessment stability of both scales is evaluated by test-retest reliability. A statistical approach will be applied that is suitable for all types of data having at least an ordered structure, though distances and magnitude are unknown [17].

## Methods

### Subjects

Outpatients with diagnosed pain conditions were consecutively recruited from the rehabilitation medicine clinic and the spinal cord injury out patient department at the Karolinska University Hospital, in Stockholm. The assessments were conducted in accordance with the declaration of Helsinki and the patients gave their informed consent to participate. The study was approved by the Ethics Committee of Karolinska University Hospital (dnr 03–162).

The patient's pains, in general located to, and/or projected to the musculoskeletal system, were previously classified according to its etiology by their physicians into – chronic/idiopathic pain, nociceptive or neuropathic pain [12,13]. The chronic/idiopathic pain was described as generally persistent, distributed without neuro-anatomical distribution and present without noxious stimulus which could result from abnormal processing of normal input in the central nervous system. The criteria of nociceptive pain can be described as a response to activation of damaged tissue where the local pain intensity increases during movement or loading of the affected tissue. The characteristic features of neuropathic pain were among the patients in this study, pain located at and/or below the level of the damaged neural structure, i.e. in this case the spinal cord injury, in an area with altered sensibility and persistent or spontaneous pain unrelated to loading.

All patients were also asked about their prescription of analgesics and whether they had consumed any pain killing drugs on the day of assessment.

### Study design and pain rating scales

This is a cross-sectional study in the sense that the three pain etiology groups will be described separately. In order to avoid assessment bias the two scales for self-rated pain intensity were administrated to the patients in random order 30 minutes prior to their appointment, scheduled in advance, with their physician. The scales were a continuous, horizontal, visual analogue scale, VAS, (0–100) with the anchor points, "no pain" and "worst possible pain " respectively and, a discrete, five-category, verbal rating scale, VRS, with the eligible alternatives – no pain (0), mild (1), moderate (2), severe (3), worst possible pain (4), [see Additional file 1].

Although the pain rating scales were, *per se*, familiar to almost all patients, they were again informed about their use and encouraged to try them out prior to the real assessments. Thereafter, the patients were asked to rate their actual pain intensity by marking a level on the scales corresponding to their experienced pain intensity level. In case of not perceiving pain in rest, which was the case among some of the patients with nociceptive pain, the engaged tissue was loaded by isometric muscle contractions or by testing the respective joints active/passive range of movement in order to provoke the pain and thereby be able to rate any actual pain. The VAS was presented on paper sheets and the VRS on an electronic diary (Clinitrac^®^). The assessment on the electronic diary was transformed to a code-locked data base.

The assessment procedure was repeated for the intra-individual stability evaluation. In the analyses the pain assessments on the VAS were assigned the numeric values 0 through 100 yielding 101 ordered positions.

### Statistical methods

The mean value and standard deviation (SD) were calculated for age. Frequency distributions were shown for patients' duration of pain and the use of different analgesics. The median and range (minimum to maximum) were used to describe the ordinal data of self-rated pain.

The statistical method used is designed for comparing scales with different numbers of possible response alternatives [6,7]. As each individual assessed their perceived pain on two scales the data set consists of paired data, (VAS, VRS). Interchangeability between scales with different numbers of response categories requires a high level of order-consistency, i.e. lack of overlapping of the records on one scale relative the other. A possible presence of overlapping was described and evaluated from scatter and line plots. For example, the pairs (34, no pain), (34, mild pain) and (34, moderate pain) are overlapping. The two pairs (43, mild pain) and (48, moderate pain) represent ordered pairs and the two pairs (43, severe pain) and (48, moderate pain) exemplify disordered pairs. The number of disordered pairs, out of all possible different pairs, was calculated and defines the measure of disorder, D [6,7]. The level of order-consistency is defined by the coefficient of monotonic agreement, MA, which can be calculated by MA = 1-2D and ranges from -1 to 1.

In order to describe the correspondence between condensed VAS data and the VRS categories, the continuous VAS assessments were transformed to a discrete five-category scale in two ways; the cut-off positions being defined unbiased relative the VRS assessments, and being defined equidistantly, respectively.

The cut-off positions of the visual analogue line, which define a discrete VAS that is unbiased to the VRS data, are constructed by pairing off the two sets of frequency distribution to each other and by identifying the cut-off positions in VAS that corresponds to the change in category of the VRS. This procedure creates pairs that are in complete order, MA = 1. Thus the condensed discrete scale based on the continuous VAS records will, under this circumstance, show a total order consistency and no systematic disagreement (be unbiased) relative the VRS. Another approach is to condense the continuous VAS records into an equidistant five-category scale that is to be compared with the five-category VRS.

A high level of order consistency between scales with the same number of categories, in our case the condensed VAS and the VRS, requires a high percentage agreement (PA, %) and a lack of systematic disagreement (bias) of the pairs of data. The frequency distribution of the pairs of data was evaluated by means of square (5 × 5)-contingency tables. The proportion of identical pairs defines the PA. A presence of different frequency distributions, also called marginal distributions, indicate a presence of systematic disagreement (bias), which means that the categories of the two scales have different interpretations and are, thereby, not regarded as interchangeable. Two measures of systematic disagreement were calculated; the relative position, RP, and the relative concentration, RC, with possible values ranging from -1 to 1 [16]. The RP estimates the difference between the probability of the pain assessments on one scale being shifted towards higher categories relative to the other scale and the probability of the assessments on one scale being shifted towards lower categories relative to the other. The RC estimates the difference between the probability of the pain assessments on one scale being concentrated relative to the other and vice versa.

The stability of intra-individual assessments was calculated from test-retest pairs of data and a high level of stability requires high level of intra-individual agreement, PA, and a lack of systematic disagreement, which means zero or negligible RP and RC values.

The software package of Statistica, 6.0 was used for descriptive statistics and SYSRAN 1.0 for Matlab 6 was used to calculate D, MA, RP, RC and the corresponding 95% confidence intervals for the RP and RC.

## Results

Eighty patients, (mean age 42.8; SD 12.7 years), recruited from the three pain groups, participated in the study and rated their actual pain intensity. All were capable to independently managing the pain assessment instruments. Analgesic drugs were most frequently prescribed to the patients in the chronic/idiopathic and neuropathic pain groups. On the day for pain intensity assessments, fewer patients had consumed analgesic drugs than what was prescribed, table 1.

**Table 1:** Demographic data of pain patients.

	Pain etiology group		
	Chronic/idiopathic, n = 30 (women, n = 13)	Nociceptive, n = 31 (women, n = 15)	Neuropathic, n = 19 (women, n = 8)
Age, mean (SD), years	42.8 (10.6)	40.0 (14.2)	47.3 (12.7)
Duration of pain, months, n (%)			
0–3		8 (26)	1 (5)
4–6		6 (19)	
7–12		5 (16)	3 (16)
> 12	30 (100)	12 (39)	15 (79)
Patients prescribed with analgesics, n (%)	26 (87)	3 (10)	17 (89)
Patients consuming analgesics the day of assessments, n (%)	11 (37)	3 (10)	14 (74)

The results of the first assessments were chosen for inter-scale comparison and both assessments were used for the test of intra-scale stability.

**Figure 1:**
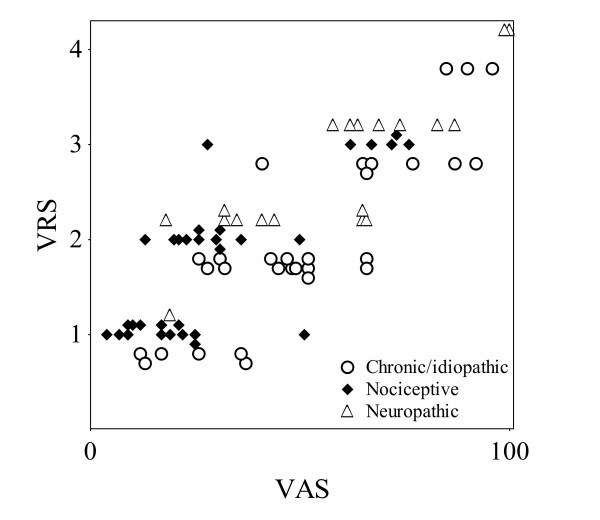
Joint distribution of rated pain intensity on the continuous VAS versus the discrete VRS in patients with chronic, nociceptive and neuropathic pain, respectively.

**Figure 2:**
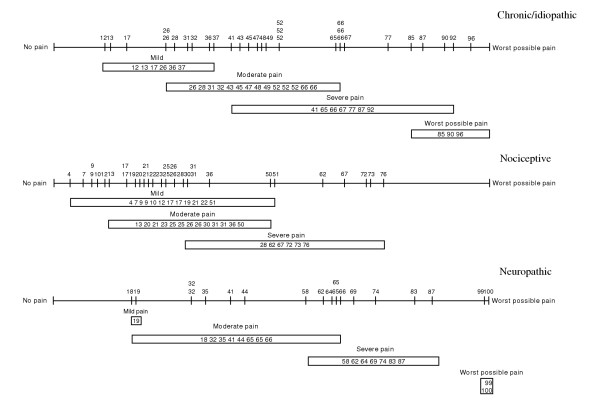
Line plots of recorded rated pain intensity on continuous VAS, 0–100 and on the VRS relative the VAS, for the three pain etiology groups respectively.

The median levels of rated pain intensity on the VAS were: chronic/idiopathic pain, 59 (range, 12 to 96); nociceptive pain, 25 (range, 4 to 76); neuropathic pain 64 (range, 18 to 100), fig 2. The corresponding median levels of VRS ratings were moderate (2) for all subgroups but with different ranges: chronic pain ranged from mild pain (1) to worst possible pain (4); nociceptive pain ranged from mild pain (1) to severe pain (3); neuropathic pain ranged from mild pain (1) to worst possible pain (4), figure 1.

### Inter-scale comparison, continuous VAS versus VRS

Overlapping VAS records relative the VRS categories mild, moderate, and severe pain were seen in all groups in this study, figures 1, 2, indicating that rated pain intensity labeled as e.g. moderate and severe according to the VRS corresponds to any possible value from 26 to 66, and from 41 to 92 respectively on the VAS in chronic/idiopathic pain patients.

The measured level of concordance, monotonic agreement (MA), was found to be similar in all groups of etiology (chronic/idiopathic pain, MA = 0.89; nociceptive pain, MA = 0.87; neuropathic pain, MA = 0.88), revealing a difference between the ordered and disordered pairs of assessments.

### Inter-scale comparison, discrete VAS versus VRS

**Figure 3:**
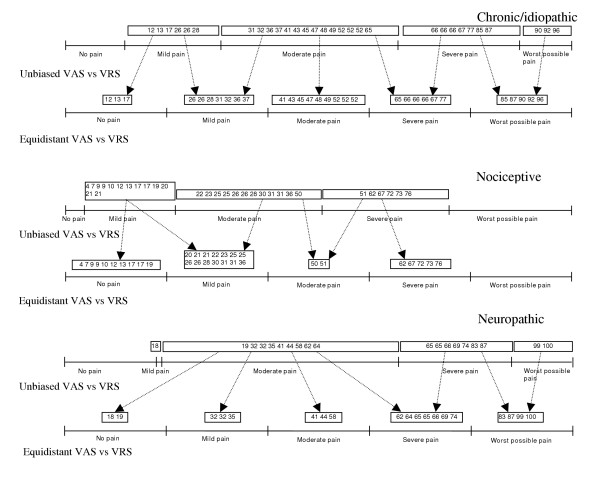
Line plots of VAS records condensed into discrete five-category scales relative the VRS – totally ordered (unbiased) and equidistant for the three pain etiology groups respectively.

The cut-off positions of the discrete five-category VAS, unbiased to the VRS, were similar in the chronic/idiopathic and neuropathic pain groups (12, 31, 66, 90 and 18, 19, 65, 99 respectively) while cut-off positions in the nociceptive pain group were lower (4, 22, 51, 77), figure 3. The different cut-off positions indicate that the rated pain intensity could have different meaning depending on pain etiology. Figure 3 also shows the inconsistencies between the scales when the VAS records were divided into a five equidistant category scale. For example a patient rating the perceived pain as mild, could be labeled as no pain in the equidistant VAS. This phenomenon was seen in all groups.

**Figure 4:**
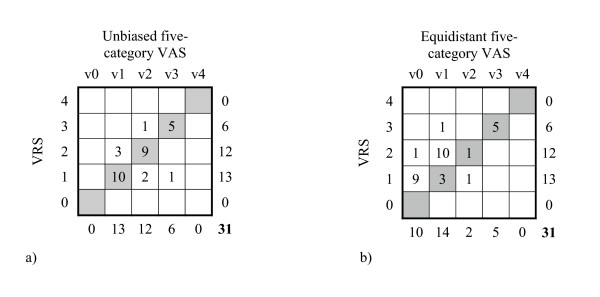
a–b Contingency tables of frequency distribution of discrete VAS records relative the VRS on a) the unbiased five-category VAS (v0–v4) relative the VRS (0–4) and b) the equidistant five category VAS (v0–v4) versus the discrete five category VRS (0–4) in patients with nociceptive pain. Agreeing pairs of data are shown in the grey shaded main diagonal.

The observed inconsistencies between the scales imply lack of interchangeability which were confirmed by the PA (ranging from 29% to 60%) and the measures of systematic disagreement, especially in concentration (RC), figure 4a–b, table 2.

**Table 2:** Inter-scale comparisons of five categories VAS versus VRS.

	Unbiased VAS vs VRS		Equidistant VAS vs VRS			
Pain etiology group	PA (%)	MA	PA (%)	MA	RP (95% CI)	RC (95% CI)
All, n = 81	73	0.93	44	0.96	0.18 (0.07 to 0.28)	0.43 (0.33 to 0.53)
Chron/idiop, n = 30	67	0.95	60	0.99	0.06 (-0.07 to 0.20)	0.27 (0.12 to 0.42)
Nociceptive, n = 31	77	0.90	29	0.91	0.44 (0.27 to 0.61)	0.56 (0.29 to 0.83)
Neuropathic, n = 19	58	0.86	42	0.96	-0.02 (-0.25 to 0.21)	0.36 (0.08 to 0.64)

Chron/idiop = Chronic/Idiopathic; PA = Percentage Agreement; MA = Monotonic Agreement; RP = Relative Position; RC = Relative Concentration; CI = Confidence Interval

### Test-retest reliability, intra-scale stability

In the two repeated VAS assessments a low proportion of the patients, 11% to 26%, in the three groups recorded the same pain level, and 87% to 100% of the patients recorded the same level in the repeated ratings on the VRS, table 3.

**Table 3:** Test of intra-scale stability in VAS (0–100) and VRS.

	VAS			VRS		
Pain etiology group	PA (%)	RP (95% CI)	RC (95% CI)	PA (%)	RP (95% CI)	RC (95% CI)
All, n = 81	20	0.01 (-0.03 to 0.04)	-0.02 (-0.08 to 0.05)	94	0.03 (-0.01 to 0.06)	0.04 (-0.01 to 0.08)
Chron/idiop, n = 30	20	-0.05 (-0.12 to 0.01)	-0.07 (-0.18 to 0.03)	97	0.02 (-0.02 to 0.07)	-0.01 (-0.04 to 0.02)
Nociceptive, n = 31	26	0.07 (-0.004 to 0.15)	0.03 (-0.05 to 0.12)	87	0.06 (-0.04 to 0.16)	0.11 (-0.01 to 0.22)
Neuropathic, n = 19	11	0.04 (-0.06 to 0.14)	-0.05 (-0.19 to 0.10)	100	0.00	0.00

PA = Percentage Agreement; RP = Relative Position; RC = Relative Concentration; CI = Confidence Interval

## Discussion

The results of this study showed overlapping records between the two scales and a comparable level of inter-scale discordance in all pain etiology groups. For the VAS data condensed into a discrete scale unbiased the VRS, the cut-off positions corresponding to the labels – no pain, mild, moderate, severe and worst possible pain – were similar in patients with chronic/idiopathic and neuropathic pain but lower in the patients with nociceptive pain, indicating influence depending on pain etiology. For the equidistant discrete VAS data a systematic disagreement especially in concentration was found relative the VRS levels which means lack of interchangeability. Similar consequences of condensing continuous VAS data into discrete levels have been found elsewhere [6,7] but is not discussed in the findings of Jensen et al. [18].

In the test-retest of the two scales, a low percentage agreement were seen in assessments on VAS through all pain categories, where only 11 to 26% of the patients agreed to the same level, while a high percentage agreement were found in assessments on VRS where 87 to 100% of the patients agreed to the same level. No systematic disagreement was found in test-retest of either scale. The results of this study therefore imply that the records of self-assessed pain intensity on the VAS and the VRS, performed by the same individuals, are not interchangeable, possibly requiring different interpretation, and that the pain intensity assessments on the VAS do not have linear properties. This confirms the results of Svensson and Svensson et Berndtson [6,7,9] in evaluating the use of rating scales for the assessment of subjective variables.

The lack of operational definition of the VAS can possibly induce insecurity on how to relate to the continuous VAS line, thereby contributing to the low percentage agreement of the individual repeated records. The principles of pain classification, that are continuously discussed, could also contribute to the variable results of the present study. Due to its complexity, the pain classifications are not easily executed and there may be unidentified differences between the different pain etiologies, i.e. chronic/idiopathic, nociceptive and neuropathic pain. For instance, the chronic/idiopathic pain is not recommended to be regarded as a single entity [13] since it may include several etiologies and, furthermore, may be referred to as a disease on its own rights [19]. The associated chronic pain is probably not directly related to their initial injury or disease condition, but rather to secondary changes, including ones that occur in the pain detection system itself [14,19]. Also the diversity in neuropathic pain conditions is discussed in terms of its appearance as "definite, possible or unlikely" [20] and, besides the existence of varying degrees of 'neuropathic' components in chronic pain conditions [20,21]. According to the classification by Rasmussen et al. [20], the patients in this study that were classified as neuropathic, could with most certainty be considered as definite since the etiology is spinal cord injury. The patients suffering from chronic pain may, on the other hand, include some degree of possible neuropathic pain. The pain experience may also be influenced by multiple other factors such as gender, cultural conditioning, expectations, social contingencies, mood state, and perceptions of control. In a future however, the principles of pain categorization is hypothesized to be based on the pain mechanism [14].

There is a controversy in the literature regarding which rating scale being most sensitive to change. Because verbal scales usually have few steps, they are considered to be less sensitive than VAS. Breivik and collaborators [8] reported that assessments of acute pain with a four category VRS, was less sensitive than VAS, 0–100, while VAS and an eleven category NRS, showed similar sensitivity and was recommended to be adopted based on subjective preference. Interestingly the VAS scores were, in the same study, reported as being possible to be classified into any of the four VRS categories. Furthermore, the shortcomings of using the VRS has been described as that the patient is forced to translate a feeling into a predefined word that possibly not fit exactly to the patient's experience and, also, that the same word does not necessarily mean the same thing to each patient [4]. On the other hand, a recent study showed that a VRS was superior to the VAS, NRS, verbal numerical rating scale and a faces pain scale considering internal consistency reliability, sensitivity, and preferred by adults [22]. Furthermore, a preference for VRS over VAS was found by Clark and collaborators [23] when 113 patients were asked, and the VRS is recommended for clinical trials due to it easiness to learn how to handle and to interpret its changed score [24].

Consistent with the findings of Ponce de Leon et al. [25], we found a greater intra-individual agreement using the VRS than using the VAS for assessment of subjective phenomena such as pain. The reason for this response may be due to the use of verbal descriptors or the use of only five categories, but also possibly due to that subjective perceptions, such as pain, could be more easily expressed in words than by a mark on a continuous line without operational definition or by numbers. Different expressions such as faces and images could also serve as response alternative of perceived pain level.

Based on the results of this study, numerals in pain rating seem meaningless since rated moderate pain intensity could be presented on the VAS with a range from 22–65 though there are suggestions of regarding ratings more than 30 mm on VAS as probable moderate and ratings more than 54 mm as probable severe when using a 4-point categorical scale [26].

### Limitation of this study

One limitation of our study could be the small number of patients and the possible presence of various pain etiologies in some individuals. Our results refer to rated, individual actual pain intensity of patients suffering from pain of different etiologies and cannot be generalized to other situations.

## Conclusion

The records of actual pain intensity on the VAS and the VRS are, in this study, not interchangeable in any of the pain etiology groups due to overlap of pain records between the two scales, systematic disagreements when comparing the two scales and a low percentage intra-scale agreement. Furthermore, the lower VAS cut-off positions relative the VRS labels indicate different meaning of the rated pain intensity depending on pain etiology. The results also indicate that the scales have non-linear properties and that the two scales probably have different interpretation. Our findings are in favor of using the VRS in pain intensity assessments but if still the VAS is preferred, the VAS data should be analyzed as continuous using statistical methods suitable for ordinal data. Furthermore, our findings indicate a risk to over or under estimate the patient's perceived pain when interpreting condensed VAS data.

## Competing interests

The author(s) declare that they have no competing interests.

## Authors' contributions

IL, TL, JK, CNB and LS designed the study. CNB and LS collected the data. IL extracted and analyzed the data. JK designed and supplied the electronic diaries. IL wrote the manuscript and TL and ES critically revised different sections of the manuscript. All authors contributed to commenting on drafts of the manuscript and have read and approved the final manuscript.

## Pre-publication history

The pre-publication history for this paper can be accessed here:



## Supplementary Material

Additional File 1 Supplementary Figure 1a-b. The two rating scales used for self-assessed actual pain intensity. In the analysis, the VAS and the VRS assessments were assigned the numeric values 0 through 100 and 0 through 4 respectively, each with the anchor points "no pain" and "worst possible pain" respectively. Click here for file
