# Supplementary material for: Lack of interchangeability between visual analogue and verbal rating pain scales: a cross sectional description of pain etiology groups
Source: BMC Med Res Methodol. 2005 Oct 4;5:31. doi: 10.1186/1471-2288-5-31 (PMC1274324; doi:10.1186/1471-2288-5-31)
Supplement: Additional File 1 — Supplementary Figure 1a-b. The two rating scales used for self-assessed actual pain intensity. In the analysis, the VAS and the VRS assessments were assigned the numeric values 0 through 100 and 0 through 4 respectively, each with the anchor points "no pain" and "worst possible pain" respectively. [file 1471-2288-5-31-S1.doc]

**a) The visual analogue scale, VAS**

”How intense do you perceive your actual pain?”

No pain

Worst possible pain

**b) The verbal rating scale, VRS**

”How intense do you perceive your actual pain?”

⭘ No pain

⭘ Mild pain

⭘ Moderate pain

⭘ Severe pain

⭘ Worst possible pain
